# Noninvasive quantitative assessment of collagen degradation in parchments by polarization-resolved SHG microscopy

**DOI:** 10.1126/sciadv.abg1090

**Published:** 2021-07-16

**Authors:** Margaux Schmeltz, Laurianne Robinet, Sylvie Heu-Thao, Jean-Marc Sintès, Claire Teulon, Guillaume Ducourthial, Pierre Mahou, Marie-Claire Schanne-Klein, Gaël Latour

**Affiliations:** 1 Laboratoire d’Optique et Biosciences, Ecole polytechnique, CNRS, INSERM, Institut Polytechnique de Paris, Palaiseau, France.; 2 Centre de Recherche sur la Conservation (CRC), Muséum national d’Histoire naturelle, Ministère de la Culture, CNRS, Paris, France.; 3 Université Paris-Saclay, Saint-Aubin, France.

## Abstract

Nondestructive and noninvasive investigation techniques are highly sought-after to establish the degradation state of historical parchments, which is up to now assessed by thermal techniques that are invasive and destructive. We show that advanced nonlinear optical (NLO) microscopy enables quantitative in situ mapping of parchment degradation at the micrometer scale. We introduce two parameters that are sensitive to different degradation stages: the ratio of two-photon excited fluorescence to second harmonic generation (SHG) signals probes severe degradation, while the anisotropy parameter extracted from polarization-resolved SHG measurements is sensitive to early degradation. This approach is first validated by comparing NLO quantitative parameters to thermal measurements on artificially altered contemporary parchments. We then analyze invaluable parchments from the Middle Ages and show that we can map their conservation state and assess the impact of a restoration process. NLO quantitative microscopy should therefore help to identify parchments most at risk and optimize restoration methods.

## Teaser

Conservation state of medieval parchments is noninvasively assessed by advanced quantitative nonlinear optical microscopy.

## INTRODUCTION

Parchment was the main writing support material in the Middle Ages in Western Europe until the growth of paper production in the 14th to 15th centuries. It is made from untanned animal skin, which is preserved by liming, scraping, and drying under tension (*1*). After this preparation process, the main remaining constituent is fibrillar collagen, which arises from the self-assembly of collagen triple-helical molecules into fibrils and further into fibers. Parchments are very sensitive to heat and water that can induce collagen degradation. The ultimate state of degradation is the formation of gelatin that corresponds to the unfolding of the collagen triple-helical structure (denaturation) and the reorganization of the single helices into a network with small triple-helical domains (gelatin) (*2*). Such an advanced degradation can sometimes be identified macroscopically and qualitatively by a shrinkage, increased transparency, and increased rigidity of the material. As this transformation is irreversible and its primer steps are usually not visible to the eye, the main challenge for conservation scientists and restorers is to identify these early stages of degradation to avoid gelatin formation following exposure to a humid environment.

A vast collection of manuscripts on parchment of major historical relevance is housed in the library of Chartres in France. Chartres was one of the most important occidental intellectual centers during the 11th and 12th centuries, due to its invaluable collection of about 500 medieval manuscripts originating mainly from the famous Chartres chapter library. On 26 May 1944, during a bombing of the city, a fire irremediably destroyed almost half of the collection. At least 215 manuscripts survived the fire but remained in very variable states, from almost intact to entirely calcined. Considering the fragility of the fragments, a digitization campaign was started in 2006 to make these documents available again for historians (*3*). In highly deformed manuscripts, a restoration treatment was required to flatten the parchment before digitization. Since the parchments had suffered from highly damaging conditions, exposure to a moisture treatment to relax the collagen fibers might cause further damage. Therefore, it is essential to assess the conservation state of the collagen before such treatment and to monitor its evolution in the most degraded parchments.

Nowadays, the degradation state of skin-based materials in the cultural heritage field is commonly assessed by differential scanning calorimetry (DSC) or by micro hot table (*4*–*6*). These thermal techniques measure the temperature, called shrinkage temperature *T*_S_, at which the fibrillar collagen turns into gelatin in a wet sample. The DSC technique also provides the enthalpy change Δ*H* related to the thermal energy required for the denaturation. However, these techniques are invasive, as they require a sampling, and destructive, which limits the number of analyzed areas and constrains them to the edges or hidden parts of the artifact rather than on the more relevant central part close to the text and illuminations. In addition, the alteration is often heterogeneous within a single parchment, so a noninvasive technique is needed to repeat the measurements in different areas of the artifact to access a complete diagnosis of the manuscript.

Noninvasive approaches have been recently proposed for parchments, for instance, to identify the animal species (*7*, *8*). Regarding the degradation state, the current analytical techniques show some limitations. Attenuated total reflection–Fourier transform infrared (ATR-FTIR) spectroscopy provides information on the extent of gelatinization in a parchment (*9*). However, it requires scraping the parchment surface before measurement to avoid interferences from surface materials, such as calcium carbonate layer or dust. Near-infrared spectroscopy can estimate the shrinkage temperature of parchments noninvasively (*10*), but needs to be calibrated with a large database of reference samples. Synchronous fluorescence spectroscopy was suggested for estimating the collagen-to-gelatin ratio, but it has not been correlated to quantitative assessment of collagen degradation such as the shrinkage temperature (*11*).

Some optical techniques provide noninvasive and nondestructive three-dimensional (3D) imaging of cultural heritage artifacts. In this respect, the most common technique is optical coherence tomography (OCT) (*12*). It has been used to study the influence of inks and pigments on the conservation state of parchments (*13*, *14*), but it is not well suited to characterize the organization of the fibers because of their dense arrangement after the manufacturing process, which homogenizes their optical properties and strongly reduces the contrast of OCT images. Alternatively, nonlinear optical (NLO) microscopy (*15*, *16*), which is widely used for biomedical studies, is today an emerging technique in cultural heritage sciences. It provides micrometer-scale 3D resolution to study pigments, binders and varnishes (*17*–*23*), paper and textiles (*19*, *24*, *25*), wood used for music instruments (*18*), and skin-based objects such as leathers, parchments, and natural history specimens (*26*, *27*). A key advantage of this technique is its multimodal capability based on different modes of contrast that are directly linked to the structural or chemical nature of the materials. Two-photon excited fluorescence (2PEF) signals are emitted by a wide range of materials (fluorophores) in historical artifacts with specific absorption and emission fluorescence spectra (*18*). Second harmonic generation (SHG) signals are specific for dense and well-aligned structures such as fibrillar collagen (*15*, *16*, *28*) and vanish for centrosymmetric materials such as gelatin. Usual SHG imaging is based on circularly polarized laser excitation, while in polarization-resolved SHG (P-SHG) microscopy, the SHG signal is recorded as a function of the orientation of the linearly polarized laser excitation. This advanced method provides the main orientation of the collagen fibrils and the degree of alignment of the triple helices within and between fibrils at the submicrometer scale, as demonstrated in various biomedical studies (*29*–*37*). We have recently shown that SHG microscopy provides structural information about the 3D organization of fibrillar collagen within parchments and other skin-based materials (*26*, *27*). Notably, this technique discriminates these two extreme states: well-preserved artifacts characterized by prevailing SHG signals related to fibrillar collagen and gelatinized ones characterized by the loss of SHG signals and the increase of 2PEF (*26*). However, basic implementation of NLO imaging does not meet the need of finely grading intermediate states of degradation, as thermal methods do.

In this study, we propose two quantitative methods based on advanced NLO microscopy to quantify these intermediate states of degradation. The first method is to compute the ratio of 2PEF to SHG signals in every pixel. The second one is to use P-SHG microscopy to compute the collagen alignment at the submicrometer scale. We first study a set of model parchments made from contemporary parchments that were artificially altered by heat exposure for increasing durations. The quantitative parameters obtained by these new methods are compared with the shrinkage temperature and the enthalpy change measured by DSC to assess their validity. Second, these quantitative methods are used to noninvasively estimate the degradation state of the heat-damaged medieval parchments from the Chartres’s library.

## RESULTS

### Model parchments

**Fig. 1:**
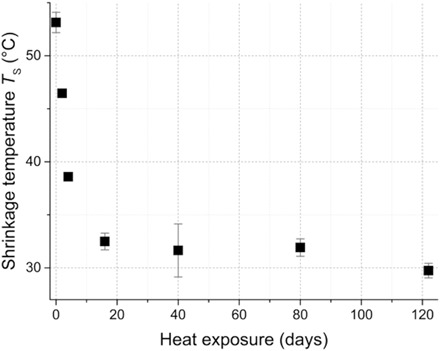
Shrinkage temperature of model parchments. Shrinkage temperature *T*_S_ obtained by DSC measurements of model parchments artificially altered by dry heat exposure during 0 (reference) to 122 days. Three sample measurements were collected for each model parchment, and the mean value and SD are displayed.

A set of model parchments D# was obtained by exposure to dry heat for # days. They were first assessed by DSC measurements of the shrinkage temperature *T*_S_ and the enthalpy change Δ*H*, as shown in Fig. 1 and fig. S1, respectively (see data in table S2). The shrinkage temperature decreases strongly from D0 (“reference” parchment) to D16 and then it stabilizes close to room temperature at around 32°C. While the shrinkage temperature *T*_S_ is constant between D16 and D122, the enthalpy change Δ*H* decreases from 41 to 5 J/g.

**Fig. 2:**
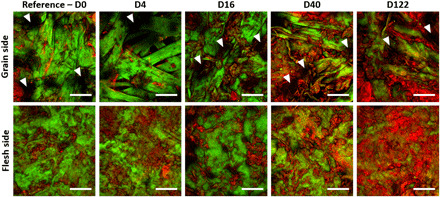
NLO imaging of model parchments. NLO imaging on grain and flesh sides of model parchments altered by dry heat exposure during 0, 4, 16, 40, and 122 days. Images in false colors: SHG signals in green and 2PEF signals in red. On the grain side, hair follicles (black holes) are shown with white arrowheads. Scale bars, 50 μm.

**Fig. 3:**
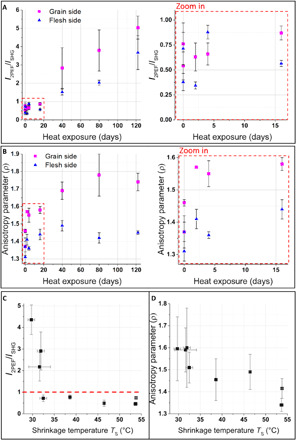
Quantitative assessment of model parchments from NLO microscopy. (**A**) Ratio of 2PEF to SHG signals *I*_2PEF_*/I*_SHG_ and (**B**) anisotropy parameter ρ on grain and flesh sides of the model parchments artificially aged by heat for 0, 2, 4, 16, 40, 80, and 122 days (one parchment for each treatment duration, but two reference parchments for D0). Zoom in (on the right): heat exposure for 0 to 16 days. (**C**) *I*_2PEF_*/I*_SHG_ and (**D**) ρ mean values from both sides of the parchments versus the shrinkage temperature *T*_S_ determined by DSC. The red dashed line in (C) corresponds to *I*_2PEF_/*I*_SHG_ = 1. Error bars correspond to the SD of measurements on several samples (*T*_S_) or several areas and sides (*I*_2PEF_*/I*_SHG_, ρ), depending on the technique (see table S1).

These model parchments were then imaged on both sides, namely, the grain and the flesh sides, by NLO microscopy. Image stacks were recorded up to a depth of 15 to 100 μm, depending on the optical properties of every parchment, to access volume information. No photodamage was observed on the samples throughout the imaging. This was assessed by verifying the reproducibility of the measurements when the same image was acquired several times in the same area. Figure 2 shows typical images merging 2PEF in red and SHG signals in green. The collagen fibers, revealed by SHG, are packed and straight, due to the stretching step during manufacturing. On the grain side, the black (no signal) round structures (white arrowheads in Fig. 2) are attributed to the location of hair follicles. On the flesh side, the collagen fiber organization seems more homogeneous and dense. In the D0 and D4 parchments, some 2PEF signals are collected from structures of few tens of micrometers, presumably due to residues of keratin, fat, and elastin, as well as gelatin induced by manufacturing (*26*). The SHG signals, arising from fibrillar collagen, prevail on the less degraded parchments, from D0 to D16, while from the D40 parchment, the 2PEF signals seem to dominate with a larger spatial extent. To quantitatively assess the evolution of these signals as a function of heat exposure duration, the ratio of 2PEF to SHG signals *I*_2PEF_*/I*_SHG_ is calculated pixel-wise and averaged in depth (see Materials and Methods) for each side of every parchment, as shown in Fig. 3A (table S3). The graph shows that this ratio does not evolve between 0 and 16 days of heat exposure and then linearly increases from 16 to 120 days.

The model parchments are further analyzed by P-SHG microscopy. It provides a measure of the anisotropy parameter ρ that is defined as the square root of the ratio of the two minima of the SHG signal as a function of the incident polarization orientation. This parameter probes the degree of alignment of the triple helices within and between fibrils in the focal volume. The expected value from previous studies on tissues is around 1.36 for well-aligned type I fibrillar collagen and increasing values are associated with increasing disorder (*32*, *34*). The evolution of the anisotropy parameter ρ versus the heat exposure duration is shown in Fig. 3B (table S3). It increases from D0 to D16 (from 1.45 to 1.58 on the grain side and from 1.3 to 1.45 on the flesh side; see the zoomed-in image in Fig. 3B), and it shows a plateau from D40. This tendency is observed on both sides of the parchments but with ρ values always higher on the grain side than on the flesh side.

To evaluate the relevance of these NLO measurements to probe the degradation state of fibrillar collagen within parchments, the NLO results are compared to the shrinkage temperature *T*_S_ and the enthalpy change Δ*H* measured by DSC. In contrast to NLO microscopy that collects signals at a selected depth between 0 and 100 μm, the DSC measurements of collagen degradation are performed over the entire thickness of the parchment (150 to 300 μm). DSC results are therefore compared to the average of the NLO data collected from the flesh and grain sides (Fig. 3, C and D). *I*_2PEF_/*I*_SHG_ is constant and remains below 1 as long as *T*_S_ is above 32°C, but this ratio increases with the decrease of the enthalpy change when *T*_S_ is below 32°C (fig. S2). On the contrary, the anisotropy parameter follows the same trend as the shrinkage temperature: in the initial stages of degradation (higher *T*_S_), it increases from 1.35 to 1.6. Moreover, for the most degraded parchments, the measurements on different regions of interest (ROIs) are strongly dispersed, which suggests that the degradation is heterogeneous within a single parchment.

### Historical parchments

**Fig. 4:**
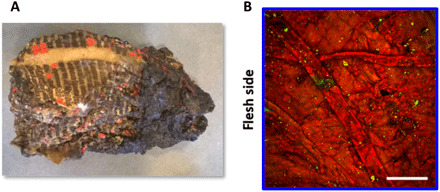
NLO imaging of a degraded medieval parchment. Investigation of a fragment from a block of agglomerated and strongly degraded parchment leaves from the Chartres’s library. (**A**) Picture of the parchment block (photo credit: M. Schmeltz, Laboratoire d’Optique et Biosciences). (**B**) NLO microscopy image on the flesh side (SHG signals in green and 2PEF signals in red). Scale bar, 100 μm.

The medieval parchments from the Chartres’s library suffered from fire and water at the end of the Second World War, as a result of a bombing and the subsequent action of the firefighters. The most degraded manuscripts appear as blocks of agglomerated parchment leaves with burned and carbonized areas, without any possibility to separate the folios (or leaves). A small and non-carbonized fragment of such a block was investigated by NLO microscopy. The collected images exhibit a compact structure with flat collagen fibers (Fig. 4). No SHG signals are collected from this sample, except from some isolated micrometric particles, likely to be calcium carbonate (*26*). The anisotropy parameter cannot be measured in this case because of the absence of SHG signals preventing any P-SHG acquisition. Anyway, low SHG signals associated to strong 2PEF signals confirm that the ultimate state of degradation of collagen has been reached in these parchments even if the morphology of some fibers is preserved.

**Fig. 5:**
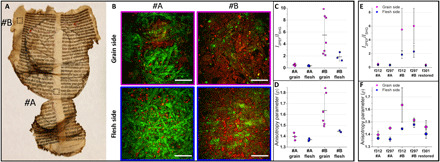
Investigation of a medieval parchment from the Chartres’s library. (**A**) Picture of the grain side of folio 312. The measurements were conducted in several ROIs located in the two areas identified by dotted squares (#A and #B) (photo credit: CNRS–IRHT). (**B**) NLO microscopy images of ROIs from both areas on grain and flesh sides of folio 312 (images in false colors: SHG signals in green and 2PEF signals in red). Scale bars, 100 μm. (**C**) Ratio of 2PEF to SHG signals *I*_2PEF_*/I*_SHG_ and (**D**) anisotropy parameter ρ for various ROIs in area #A and #B of folio 312. (**E**) *I*_2PEF_*/I*_SHG_ and (**F**) ρ for the three parchment leaves coming from the same manuscript: two leaves without treatment over 80 years (folios 312 and 297) and one leaf after restoration by cold humidification in 2009 (folio 301). Error bars correspond to the SD of measurements on several ROIs (four to six different ROIs in each area, see table S1).

Three historical parchment leaves originating from a less degraded manuscript were investigated using quantitative NLO measurements. Results for folios 312 and 297 are presented in Fig. 5 and fig. S3. Two areas are investigated on both sides: area #A, in the center, and area #B, on the edge of the folio. The central part appears less damaged by eye than the edge of the parchment, which is more rigid, shrunk, and slightly darker. Typical NLO microscopy images from these two areas on both sides are displayed in Fig. 5B. They reveal a lower SHG signal and a higher 2PEF signal in the area #B compared to the area #A, which is confirmed by the computation of the *I*_2PEF_*/I*_SHG_ ratio in all the ROIs imaged within each area (each point in Fig. 5C corresponds to one ROI and the *I*_2PEF_/*I*_SHG_ ratio is always higher in area #B). The anisotropy parameter is also higher in area #B than in area #A for all the analyzed ROIs (see Fig. 5D). In the central area #A of the folio, *I*_2PEF_/*I*_SHG_ < 1 and ρ ~ 1.4, while in the edge, area #B, *I*_2PEF_*/I*_SHG_ varies between 2 and 6 and ρ varies between 1.45 and 1.6. These results confirm the difference in collagen conservation that was visually observed between these two areas of the parchment. Similar results are obtained on folio 297 (see fig. S3).

A parchment leaf (folio 301) originating from the same manuscript and restored in 2009 was also analyzed to evaluate the influence of the restoration treatment on the collagen degradation state. The parchment had been relaxed by cold humidification to be flattened. Although it improved the visual appearance and the reading of the parchment, this water addition may have induced a degradation of the collagen. The anisotropy parameter values obtained by P-SHG measurements in the central part of the restored folio are similar to those obtained in the center of the two untreated folios from the same manuscript, as shown in Fig. 5 (E and F) and fig. S4.

## DISCUSSION

In the field of cultural heritage, one of the challenges is to develop noninvasive and nondestructive approaches that provide a precise diagnosis related to the conservation state and the type of degradation of the historical parchments. The two main chemical degradation mechanisms taking place in collagen are hydrolysis and oxidation (*1*). Both reactions cause chain cleavage with the formation of peptides, and the modification of the collagen side group chemistry, leading to alteration of hydrogen bonding and other interactions stabilizing the triple-helical structure and the fibrillar assemblage (*4*). Water access is thus facilitated to further break hydrogen bonds and unfold the collagen triple helices. As the stabilizing forces decrease, the energy required for denaturation of the collagen molecule decreases. This denaturation and the concurrent formation of gelatin correspond to an irreversible degradation.

Currently, collagen stability is assessed by measurement of the shrinkage temperature from a parchment sample, using destructive thermal techniques. In contrast, NLO microscopy is a noninvasive and nondestructive technique for investigating the conservation state of parchments. No variation of the morphology of the collagen fibers and no modification of the SHG and 2PEF signals were detected over time during the image acquisition. It proved the absence of any degradation of the fibrillar collagen due to laser heating during parchment imaging since any damage would have induced a variation of the SHG and 2PEF signals.

In the heat-damaged model samples presented in this study, the shrinkage temperature *T*_S_ decreases sharply until 16 days of heat exposure, down to its limit, around 30°C. At this state of degradation, contact with water at ambient temperature is sufficient to turn the remaining fibrillar collagen into gelatin (*4*). Beyond 16 days of heat exposure, the shrinkage temperature remains constant but the enthalpy change decreases, which may indicate that the amount of preserved fibrillar collagen within a sample diminishes and therefore that the degradation, initially localized on the parchment surface, extends into the material depth. Together, these measurements clearly confirm that the collagen degradation increases with heat exposure duration and that the parameter *T*_S_ is sensitive to the first steps of degradation of the fibrillar collagen fragilized by chain cleavage.

Thanks to the correlation established between DSC and NLO quantitative parameters on the model parchments, we propose a noninvasive alternative to quantify the collagen degradation state in parchments. The SHG signals collected by NLO microscopy in biological tissues are specific for dense non-centrosymmetric assemblies of aligned peptide bonds (*15*, *16*, *28*), as in fibrillar collagen, where the α chains are tightly aligned within triple helices, which are, in turn, tightly aligned within fibrils. In contrast, gelatin (denatured collagen) forms a centrosymmetric and low-density network of single helices, connected by small triple-helical domains, and accordingly, it exhibits no SHG signal (*26*). During collagen degradation, SHG signal is thus lost because of the alteration of the collagen hierarchical structure. Concomitantly, 2PEF intensity increases until it predominates. The origin of the 2PEF intensity observed in degraded collagen is still unknown. Infrared spectroscopy showed the onset of a carbonyl band during this transformation, which was previously attributed to the formation of gelatin (*26*). In the present study, the heat treatment applied to the model parchments induces oxidation of the collagen with breakage of the N─C covalent bond within the main chain and cross-linking of the molecules (*6*, *38*). However, the preservation of the fibrillar morphology in some of the heat-damaged parchments suggests that the increase of the 2PEF intensity is not specific to gelatin formation, but is rather a general marker of collagen degradation. In the model parchments, the ratio of 2PEF to SHG signals *I*_2PEF_*/I*_SHG_ remains stable below 1 in the first stages of the degradation. Then, *I*_2PEF_*/I*_SHG_ increases at the latest stages of degradation when *T*_S_ has reached its lowest level, around 32°C, and the degradation likely progresses in depth, as supported by the decrease of the enthalpy change. Our results thus demonstrate unambiguously that *I*_2PEF_*/I*_SHG_ is a marker for the latest stages of degradation. The measurement of *I*_2PEF_*/I*_SHG_ values above 1 (meaning that 2PEF intensity is predominant over SHG signal) in a parchment is therefore a probe of shrinkage temperature close to ambient conditions. It could thus be used to assess parchments in advanced stages of degradation, so most at risk.

The anisotropy parameter is instead sensitive to the first stages of parchment degradation as the DSC shrinkage temperature *T*_S_. The anisotropy parameter probes the submicrometer orientation disorder of collagen triple helices between and within fibrils lying within the image plane (*32*, *34*). The lower this parameter, the greater the collagen alignment within the focal volume. The increase of the anisotropy parameter is thus attributed to a disorganization of the fibrils induced by heat exposure. In the early stages of degradation, this disorganization is not sufficient to reduce the SHG signal, as revealed by the absence of evolution of *I*_2PEF_*/I*_SHG_, but it can be detected by the P-SHG response, thanks to the high sensitivity of this polarimetric technique to orientation disorganization (*32*–*34*, *37*). The anisotropy parameter is therefore complementary to the *I*_2PEF_*/I*_SHG_ value, as it provides measures of the early stages of the collagen degradation in parchment. This parameter would be particularly useful to investigate the impact of a specific environment or conservation treatment on the parchment microstructure.

Besides the state of alteration, we demonstrate that the two NLO quantitative parameters are also sensitive to some important parchment features. The measurements of the anisotropy parameter revealed a difference in the submicrometer-scale fibril distribution between the grain and the flesh sides. This difference may be attributed to the collagen organization in skin (*26*, *27*): small and disorganized fibers around hair follicles on the grain side (higher ρ values) in contrast to greater and well-aligned fibers on the flesh side (lower ρ values). Furthermore, the *I*_2PEF_*/I*_SHG_ is always higher on the grain side compared to the flesh side (Fig. 3A). This is consistent with the presence of smaller fibrils and hair follicles, as well as the presence of higher fat content on the grain side that facilitates the degradation, in contrast to the denser organization on the flesh side. In addition, thanks to the high resolution of the technique, the *I*_2PEF_*/I*_SHG_ and ρ measurements are sensitive to the heterogeneity of degradation states in the analyzed areas. The heterogeneity of the most damaged areas of the parchments is endorsed by the observation of broader DSC peaks as well as the greater error bars of the *I*_2PEF_*/I*_SHG_ and ρ measurements [20.6% for *I*_2PEF_*/I*_SHG_ (2.9% for ρ) in the D40 to D122 parchments versus 16.9% for *I*_2PEF_*/I*_SHG_ (1.8% for ρ) in the D0 to D16 parchments].

The parchment manuscripts that survived the disaster in the Chartres’s library in 1944 are a precious testimony of the past that aroused the interest of many researchers and historians. This collection is of great cultural and historical importance, as it not only contains records of description of the regional society organization in the Middle Ages but also includes a large number of Carolingian manuscripts (8th to 10th centuries), major witnesses to literary works from the Antiquity and the high Middle Ages. Considering the extreme conditions they experienced, the conservation state of these parchments is an important issue, particularly when conservation treatments are considered to improve their legibility. Most parchments recovered from the fire display visually heterogeneous degradation states even at the scale of a parchment folio. The study of these historical parchments confirms that quantitative NLO is an efficient method to assess and compare the conservation state of collagen in various parchments or within a single leaf, especially in central relevant parts near the text and the illuminations. It enables the identification of intermediate states of degradation: The edges, more exposed, are more degraded than the center of the parchment folios. Thanks to the correlations established between the *I*_2PEF_*/I*_SHG_ or ρ values and the *T*_S_ measurements on the model parchments, one can assess the conservation state of the collagen in these historical parchment leaves. In the folio edges, *I*_2PEF_*/I*_SHG_ > 1 and ρ values between 1.45 and 1.6 suggest that the shrinkage temperature of the collagen is very low in that area, and probably close to ambient temperature. In contrast, in the central part, *I*_2PEF_*/I*_SHG_ < 1 and ρ ≈ 1.4 suggest that the *T*_S_ is much higher. In heavily damaged parchments with low *T*_S_, the supply of water even through humidification may be sufficient to turn the collagen into gelatin at ambient temperature. The absence of alteration following the humidification treatment in the central area of the restored parchment thus supports our inference of a high *T*_S_ in the central part. Overall, this study of the Chartres’s manuscripts on parchment demonstrates that advanced NLO microscopy can quantitatively and noninvasively assess the degradation state of historical parchments, and in particular before conservation treatment.

Last, the correlations that were established between DSC and NLO quantitative parameters are based on heat-damaged model parchments. In contrast, the historical parchments have been exposed to heat (fire) and subsequently water (firefighter response). Therefore, collagen in these parchments may have undergone oxidation reaction followed by hydrolysis and/or gelatinization. The consistent results obtained on the Chartres’s manuscripts thus show that NLO quantitative parameters can probe various types of degradation. Moreover, as the alteration mechanism may affect the evolution of these quantitative parameters, they may be used to reveal some behavior specific to the degradation environment. In that perspective, the morphological observation of the collagen fibers with NLO microscopy may provide additional information related to the origin of the degradation. For instance, previous studies based on observation under a light microscope (*6*) have shown that the flat fiber morphology, also observed by NLO microscopy for the most damaged medieval manuscript (Fig. 4), is characteristic of oxidation by dry heat. This could be assessed quantitatively using texture analysis or other morphological parameters, as a complement to *I*_2PEF_*/I*_SHG_ and ρ. Therefore, the possibility to access quantitative information on alteration mechanism in a noninvasive way opens up new interesting research perspectives on the degradation of parchments.

In conclusion, this study has established advanced NLO microscopy as a nondestructive and noninvasive technique for the quantitative diagnosis of collagen degradation in historical parchments. The methods we have developed can be implemented on a commercial NLO setup, provided the few improvements detailed in Materials and Methods, so that they could be transferred to heritage scientists in museums. Measurements of the ratio *I*_2PEF_*/I*_SHG_ could help to identify parchments most at risk, close to the irreversible collagen denaturation into gelatin. The anisotropy parameter ρ obtained from P-SHG measurements, which probes the earlier stages of degradation, could be used for collection follow-up, thus helping heritage professionals to fine-tune conservation conditions, prevent any degradation onset, and check the collagen preservation during a restoration treatment. As a further advantage, these noninvasive NLO measurements could be repeated in different areas of an artifact to assess heterogeneous progress of degradation or in the most valuable near-text and illumination areas. Last, this new method could be applied to other collagen-based materials found in museums such as leather, natural history specimens, or fluid collections, and extended to cellulose-based artifacts, which also exhibit SHG signals, such as paper, textiles, and wooden objects.

## MATERIALS AND METHODS

### Model samples

Contemporary “model” parchments were made in 2005 out of calf skin for the European research project IDAP (Improved Damage Assessment of Parchment) (*38*). In this work, we studied the parchments subjected to artificial aging by dry heat in an oven at 100°C for various durations: 0 (“reference” parchment), 2, 4, 16, 40, 80, and 122 days. There were two reference parchments and one parchment for every other condition, which means eight parchments in total. Each sample is identified by its number of days of aging (for instance, the sample D4 corresponds to the parchment that underwent 4 days of heat exposure).

### Chartres’s medieval manuscripts on parchment

Medieval manuscripts on parchment from the Chartres’s library in France were partially destroyed at the end of the Second World War in 1944, exposing its collection to fire and then to water. Following this disaster, all recovered parchments were washed in a bath containing water and formaldehyde to clean and prevent further microbial attack. The studied corpus includes a fragment of an agglomerated parchment and three parchment leaves from the same manuscript 205, a theological compendium dating from the 13th century [folios 297 (*39*), 312 (*40*), and 301 (*41*)]. The three folios thus underwent the same degradation conditions, but folios 297 and 312 did not experience any restoration treatment over 80 years, whereas folio 301 was treated in 2009 by cold humidification to flatten the parchment.

### Differential scanning calorimetry

A PerkinElmer DSC 8000 calorimeter was used after calibration for temperature and heat flow with indium and dodecane standards. Parchment samples of around 1 mg were immersed in deionized water for 1 hour at low temperature (around 5°C). Wet samples were then placed in sealed 30-μl aluminum capsules (resistant up to 3 bars pressure) and analyzed by DSC, where they were heated from 5° to 120°C at a speed of 10°C/min. The measured thermograms displayed an endothermic peak when the collagen from the sample turned into gelatin. The shrinkage temperature *T*_S_ was measured at the onset of the peak, determined as the intersection of the peak rising slope with the baseline in the thermogram, and the enthalpy change per unit mass Δ*H* was obtained from the integration of the surface under the peak (fig. S1A) (*5*). For each model parchment, three samples were collected, measured, and lastly averaged (see table S2).

### NLO microscopy

A custom-built laser scanning upright microscope was used to perform the NLO measurements (*42*). The excitation source was a femtosecond titanium:sapphire laser (MaiTai, SpectraPhysics) tuned to 860 nm, delivering 100-fs pulses at an 80-MHz repetition rate, and scanned in the imaging plane using galvanometric mirrors. The excitation power was 5 to 10 mW at the focus. A shutter was placed before the scanning system and opened only while acquiring, to limit the exposure time and to prevent photodamages. A high–numerical aperture (NA) air objective (20×, NA 0.80, Plan-Apochromat, Zeiss) was used. This type of air objective is coverslip-corrected for biomedical studies. To compensate for this coverslip correction and achieve the best possible resolution, a resin cap with a glass lamella on its top was mounted on the objective (see fig. S5). With this configuration, the experimental resolution was 0.6 μm in the lateral direction and 1.85 μm in the axial direction near the sample surface (*43*). 2PEF and SHG signals were detected using suitable spectral filters (FF01-720/SP and FF01-680/SP from Semrock, and GG455 from Schott for the 2PEF signal and FF01-720/SP, FF01-680/SP and FF01-427/10 from Semrock for the SHG signal) and photon-counting photomultiplier tubes (P25PC, Sentech). Epidetection was used as the only configuration compatible with nontransparent parchments or thick books and manuscripts. In all the figures, 2PEF and SHG signals are represented in false colors (in red and green, respectively).

### P-SHG microscopy

P-SHG was performed using two motorized achromatic waveplates inserted at the back pupil of the objective to achieve well-defined tunable linear polarizations, with a residual field ellipticity less than 5% (*42*) (see fig. S6). 3D data were recorded by axially shifting the objective mounted on a translation stage with 1-μm axial steps, up to 15 to 100 μm depending on the penetration depth of each sample. Four to six image stacks for each side of each parchment were recorded, i.e., 67 3D stacks for the model parchments and 34 3D stacks for the historical parchments (see fig. S7 and table S1).

### Image processing and analyses

Two parameters were extracted pixel-wise from combined 2PEF/P-SHG image stacks. The parameter *I*_2PEF_*/I*_SHG_ was computed pixel by pixel as the ratio of 2PEF to SHG signals. The anisotropy parameter ρ was obtained pixel by pixel from the polarimetric diagram *I*_SHG_(θ), which is the variation of the SHG signal as a function of the incident polarization orientation θ. Details about the theoretical and numerical analysis of P-SHG data are provided in the Supplementary Materials. Briefly, ρ is defined as the square root of the ratio of the two minimal signals: $\rho =\sqrt{\frac{I_{\text{SHG}}(\varphi )}{I_{\text{SHG}}(\varphi +\pi )}}$, where φ is the position of the first minimum and corresponds to the orientation of the collagen fibril (*29*, *30*, *42*). When the collagen sample is composed of fibrils aligned within the imaging plane, as in parchments, the anisotropy parameter has been shown to probe the degree of alignment within and between these fibrils in the focal volume (*32*, *34*). It increases as the orientation disorder of the fibrils or of the triple helices within the fibrils increases at this submicrometer scale. Note that P-SHG imaging also provides the main orientation of fibrils within the focal volume, but this parameter was not of interest in this study.

A series of thresholding and averaging was then conducted to get a unique value for each parameter per parchment side (see fig. S7). The mean value and the SD of the parameters were calculated on each side from *z* stacks recorded in four to six ROIs for each model parchment and two to six ROIs in the same area for each historical parchment. All the data processing was performed using homemade routines written in Matlab or ImageJ.

## SUPPLEMENTARY MATERIALS

Supplementary material for this article is available at http://advances.sciencemag.org/cgi/content/full/7/29/eabg1090/DC1
